# Biological sex does not influence the peak cardiac output response to twelve weeks of sprint interval training

**DOI:** 10.1038/s41598-023-50016-4

**Published:** 2023-12-27

**Authors:** William Bostad, Jennifer S. Williams, Emily K. Van Berkel, Douglas L. Richards, Maureen J. MacDonald, Martin J. Gibala

**Affiliations:** 1 Department of Kinesiology, McMaster University, Ivor Wynne Centre, 1280 Main Street West, Hamilton, ON L8S 4K1 Canada; 2 Department of Medicine, McMaster University, 1280 Main Street West, Hamilton, ON L8S 4K1 Canada

**Keywords:** Physiology, Cardiovascular biology, Cardiology, Cardiovascular biology

## Abstract

Sprint interval training (SIT) increases peak oxygen uptake (V̇O_2peak_) but the mechanistic basis is unclear. We have reported that 12 wk of SIT increased V̇O_2peak_ and peak cardiac output (Q̇_peak_) and the changes in these variables were correlated. An exploratory analysis suggested that Q̇_peak_ increased in males but not females. The present study incorporated best practices to examine the potential influence of biological sex on the Q̇_peak_ response to SIT. Male and female participants (n = 10 each; 21 ± 4 y) performed 33 ± 2 sessions of SIT over 12 wk. Each 10-min session involved 3 × 20-s ‘all-out’ sprints on an ergometer. V̇O_2peak_ increased after SIT (3.16 ± 1.0 vs. 2.89 ± 1.0 L/min, η^2^_p_ = 0.53, *p* < 0.001) with no sex × time interaction (*p* = 0.61). Q̇_peak_ was unchanged after training (15.2 ± 3.3 vs. 15.1 ± 3.0 L/min, *p* = 0.85), in contrast to our previous study. The peak estimated arteriovenous oxygen difference increased after training (204 ± 30 vs. 187 ± 36 ml/L, *p* = 0.006). There was no effect of training or sex on measures of endothelial function. We conclude that 12 wk of SIT increases V̇O_2peak_ but the mechanistic basis remains unclear. The capacity of inert gas rebreathing to assess changes in Q̇_peak_ may be limited and invasive studies that use more direct measures are needed.

## Introduction

Sprint interval training (SIT) is typically characterized by brief, intermittent bursts of ‘all-out’ or near-maximal efforts at workloads equal to or greater than the pace that elicits peak oxygen uptake (V̇O_2peak_)^1–3^. SIT protocols involving training sessions that last ≤ 15 min including recovery periods can increase V̇O_2peak_ when performed several times per week over multiple weeks^4^. V̇O_2peak_ reflects the peak integrated capacity of many physiological processes including ventilation, pulmonary oxygen diffusion, cardiac output (Q̇), circulatory oxygen delivery, muscle oxygen diffusion, and muscle oxygen use^5^. According to the Fick principle, changes in V̇O_2peak_ are broadly attributed to changes in peak cardiac output (Q̇_peak_) and/or peak arteriovenous difference in oxygen content (peak a- vO_2diff_)^6^. The mechanistic basis for SIT-induced increases in V̇O_2peak_ is unclear. This lack of understanding is likely owing to factors including the duration of training interventions, the time course for potential changes in specific physiological variables that affect V̇O_2peak_, as well as the methods used to assess these variables.

Data regarding the effect of SIT on Q̇_peak_ are limited and equivocal. SIT-induced increases in V̇O_2peak_ have been reported after 2–4 wk of SIT without a measurable change in Q̇_peak_^7,8^, and there are conflicting data regarding the Q̇_peak_ response to 6 wk of SIT^9,10^. We recently reported an increase in Q̇_peak_, measured using inert gas rebreathing (IGR), after 12 wk of SIT^11^. The change in Q̇_peak_ was also associated with an increase in V̇O_2peak_^11^. An exploratory analysis found that Q̇_peak_ increased in male but not female participants^11^, however the study was not designed using procedures deemed best practice for making sex-based comparisons^12^. Meta-analyses based on endurance training studies lasting 2–52 weeks have demonstrated greater changes in V̇O_2peak_ and cardiac remodelling in male compared to female participants^13–15^, but data regarding the potential effect of SIT are more limited. A recent review highlighted the lack of studies and conflicting evidence regarding potential sex-based differences in Q̇_peak_ trainability following SIT and the need for work to address this question using best practice research designs^16^.

The effect of SIT on other factors that can influence Q̇_peak_ is also unclear. Recent studies involving both male and female participants found that 6–8 wk of SIT increased plasma volume (PV) using a carbon monoxide rebreathe technique^10,17,18^ but others have reported no change in PV based on measurements of hematocrit (Hct) and hemoglobin concentration [Hb]^19–22^. When the blood volume of male participants was reduced through phlebotomy to that of female participants, there was no longer a difference in V̇O_2peak_ between sexes^23^. As such, potential sex differences in PV expansion following SIT may contribute to differences in Q̇_peak_ and V̇O_2peak_ trainability between males and females. The effect of SIT on endothelial function and arterial stiffness, and the potential for sex-based differences in these responses, is also relatively understudied. Endothelial function is commonly assessed by flow mediated dilation (FMD), and arterial stiffness is assessed by pulse wave velocity (PWV) and carotid artery distensibility^24–26^. A previous study^27^ reported improvements in active limb endothelial function following 6 weeks of SIT in both males and females, as assessed by using FMD of the popliteal artery, however that study did not include a sex-based comparison and there was no assessment of the clinically relevant inactive upper limb (e.g. brachial artery, in which a 1% increase in FMD is associated with a 13% decreased risk of experiencing a cardiovascular event)^28^. In contrast, endothelial function in the popliteal and brachial arteries was unchanged after 6 and 12 wk of SIT in males^29^. The discrepant results could be related to possible sex-based differences in that the group-level improvement in endothelial function in the Rakobowchuck study may have been primarily owing to the response of female participants^27^. Further, the time course of adaptation in the various arteries studied may be different^29–31^.

The primary objective of the current study was to compare changes in Q̇_peak_ between males and females in response to 12 wk of SIT. We incorporated recommended best practices for making such comparisons, including recruiting groups with similar baseline fitness relative to fat-free mass (FFM), and testing female participants during the same phase of their hormonal cycles. Secondary objectives included the effect of SIT on PV expansion and endothelial function, arterial stiffness, and carotid artery distensibility. Our primary hypothesis was that the increase in Q̇_peak_ would differ between males and females after training. Our secondary hypotheses were that there would be improvements in endothelial function following 12 wk of SIT and that this improvement would be greater in females.

## Results

### Training and descriptive data

Males and females did not differ in their baseline fitness expressed as V̇O_2peak_ relative to FFM (Table 1). Females were tested in the low hormone phase of their cycle (day 4 ± 2 of cycle) and those who were naturally cycling had an average menstrual cycle length of 28 ± 2 days. The two groups performed the same number of training sessions (M: 33 ± 2, F: 33 ± 3; *p* = 0.52) over the 12-wk intervention. Mean HR during the sprint bouts was 173 ± 10 bpm, which corresponded to 92 ± 5% of HR_peak_ elicited during the baseline V̇O_2peak_ test. Mean HR elicited over all 10-min training sessions was 150 ± 11 bpm, which corresponded to 80 ± 6% of HR_peak_. The female participants achieved a higher mean relative HR over the training session than the male participants (83 ± 7 vs. 77 ± 5% of HR_peak_; *p* = 0.02). Body mass did not change from baseline to 12 wk (77.0 ± 17 vs. 76.6 ± 16 kg, *p* = 0.59).

**Table 1:** Participant characteristics at baseline.

N	Females	Males	*P*-value
	10	10	
Age (y)	21 ± 4 [18–28]	21 ± 3 [18–28]	0.91
Height (cm)	166 ± 7 [151–176]	177 ± 7 [164–188]	**0.002**
Body mass (kg)	65.8 ± 10.5 [52.5–87.3]	88.1 ± 14.7 [68.2–109.3]	**0.001**
Fat-free mass (kg)	43.6 ± 4.9 [36.8–51.7]	65.4 ± 9.9 [47.0–79.8]	**< 0.001**
V̇O_2peak_ (L/min)	2.2 ± 0.5 [1.5–2.8]	3.6 ± 0.9 [2.5–5.2]	**< 0.001**
V̇O_2peak_ (ml/kg body mass/min)	33.2 ± 7.2 [20.3–42.8]	41.1 ± 7.3 [32.4–52.9]	**0.02**
V̇O_2peak_ (ml/kg FFM/min)	49.1 ± 7.5 [35.4–59.2]	55.1 ± 9.3 [39.0–65.5]	0.13
SBP (mm Hg)	106 ± 8 [96–125]	120 ± 9 [109–138]	**0.002**
DBP (mm Hg)	62 ± 5 [56–73]	63 ± 6 [57–72]	0.61

Data are reported as mean ± SD [range]. *p* < 0.05 between females and males are bolded.

*FFM* Fat-free mass, *SBP* Systolic blood pressure, *DBP* Diastolic blood pressure.

### V̇O_2peak_, Q̇_peak_ and associated variables

**Figure 1:**
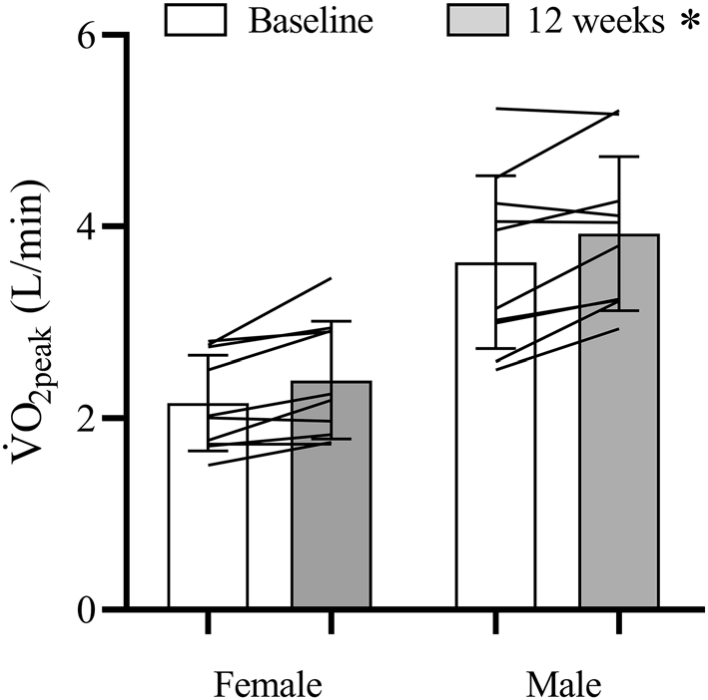
Peak oxygen uptake (V̇O_2peak_) before and after 12 wk of SIT. Mean (bars), standard deviation (error bars), and individual (connecting lines) data are presented. *p < 0.05, main effect of time.

V̇O_2peak_ increased after SIT (3.16 ± 1.0 vs. 2.89 ± 1.0 L/min; η^2^_p_ = 0.53; main effect of time, *p* < 0.001) with no sex × time interaction (η^2^_p_ = 0.014; *p* = 0.61) (Fig. 1). W_peak_ also increased with training (258 ± 74 vs. 238 ± 72 W; η^2^_p_ = 0.73; main effect of time, *p* < 0.001) with no sex × time interaction (η^2^_p_ = 0.035; *p* = 0.43). HR_peak_ was not different between the V̇O_2peak_ tests performed before and after training (188 ± 10 vs. 189 ± 12 bpm, η^2^_p_ = 0.024, *p* = 0.51) and there was no sex × time interaction (η^2^_p_ = 0.035, *p* = 0.43).

**Figure 2:**
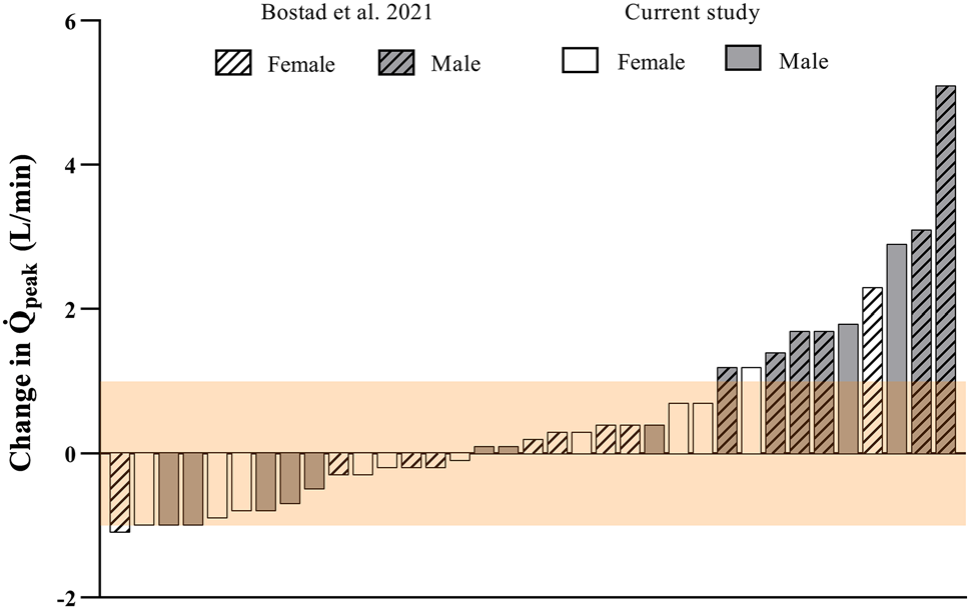
Change in peak cardiac output (Q̇_peak_) for each participant. Open and shaded bars represent the female and male participants, respectfully. Diagonal patterned bars represent participants from our previous study^11^ and clear bars represent participants from the current study. The typical error (TE) for the measurement of Q̇_peak_ (i.e., ± 1.0 L/min) is indicated by the red shaded area.

**Figure 3:**
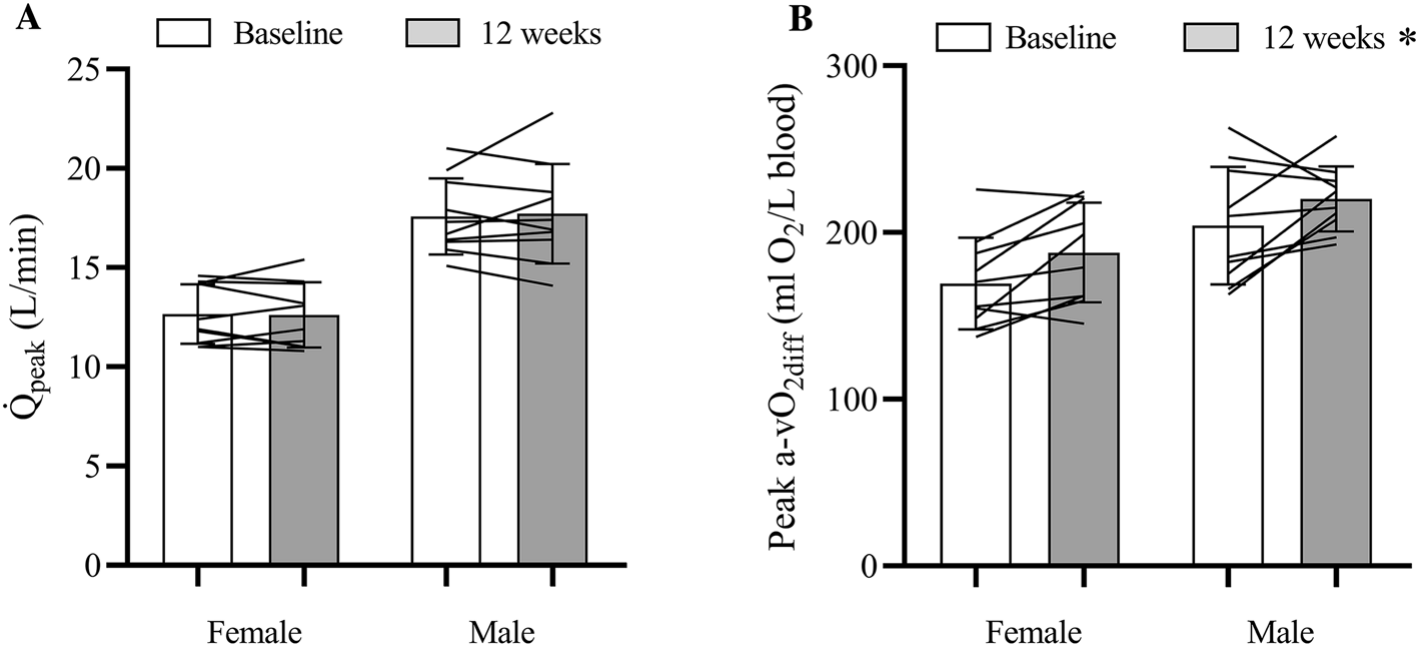
Peak cardiac output (Q̇_peak_; **A**) and peak arteriovenous oxygen difference (peak a-vO_2diff_; **B**) before and after 12 wk of SIT. Mean (bars), standard deviation (error bars), and individual (connecting lines) data are presented. *p < 0.05, main effect of time.

Q̇_peak_ was not different after SIT compared to pre-training (15.2 ± 3.3 vs. 15.1 ± 3.0 L/min, η^2^_p_ = 0.002, *p* = 0.85), and there was no sex × time interaction (η^2^_p_ = 0.007, *p* = 0.72; Fig. 3a). Individual data points for the change in Q̇_peak_ are presented in Fig. 2 and data points for Q̇_peak_ from a previous study with a similar training intervention were also included in this figure for comparative purposes^11^. Similarly, CI_peak_ was not different after SIT (8.0 ± 1.1 vs. 8.0 ± 1.0 L/min/m^2^, η^2^_p_ = 0.0004, *p* = 0.93), and there was no sex × time interaction (η^2^_p_ = 0.015, *p* = 0.61). HR_peak_ during the Q̇_peak_ test was not different at follow-up compared to baseline (185 ± 9 vs. 186 ± 10 bpm, η^2^_p_ = 0.062, *p* = 0.29) and there was no sex × time interaction (η^2^_p_ = 0.037, *p* = 0.42). Calculated peak a-vO_2diff_ increased after SIT (204 ± 30 vs. 187 ± 36 ml O_2_/L blood, η^2^_p_ = 0.35; main effect of time, *p* = 0.006; Fig. 3b), and there was no sex × time interaction (η^2^_p_ = 0.055, *p* = 0.81). V̇O_2peak_ measured by the Innocor during the Q̇_peak_ test increased after SIT (2.26 ± 0.8 vs. 2.11 ± 0.8 L/min, η^2^_p_ = 0.44, *p* = 0.001) with no sex × time interaction (η^2^_p_ = 0.099, *p* = 0.18).

### Blood data

#### Hormone analyses

Blood hormone data are reported in Table 2. Estradiol was not different at any timepoint and or between the male and female participants (*p* > 0.05 for all). There was a sex × time interaction for progesterone (η^2^_p_ = 0.23, *p* = 0.01) such that it was higher in females compared to males at wk 12 (*p* = 0.006). There was a main effect of group (η^2^_p_ = 0.94, *p* < 0.001) such that the male participants had higher testosterone levels than the female participants.

**Table 2:** Blood hormone data.

Sex hormone levels						
	Week 0	Week 4	Week 12	Time effect	Group effect	Sex × time interaction
*Estradiol (pmol/L)*						
Overall	133 ± 45	134 ± 30	126 ± 28	η^2^_p_ = 0.030 *p* = 0.59	η^2^_p_ = 0.076 *p* = 0.41	η^2^_p_ = 0.055 *p* = 0.38
Males	129 ± 39	133 ± 21	115 ± 30			
Females	138 ± 52	135 ± 38	138 ± 21			
*Progesterone (nmol/L)*						
Overall	4.3 ± 1.8	4.1 ± 1.5	4.6 ± 1.7	η^2^_p_ = 0.14 *p* = 0.08	η^2^_p_ = 0.002 *p* = 0.46	η^2^_p_ = 0.23 ***p* = 0.01**
Males	4.9 ± 1.9	4.3 ± 1.4	4.6 ± 1.8			
Females	3.6 ± 1.5	3.9 ± 1.6	4.7 ± 1.6			
*Testosterone (nmol/L)*						
Overall	8.5 ± 8.6	8.7 ± 8.2	8.4 ± 7.8	η^2^_p_ = 0.005 *p* = 0.92	η^2^_p_ = 0.94 ***p* < 0.001**	η^2^_p_ = 0.012 *p* = 0.82
Males	15.3 ± 6.3	15.9 ± 3.8	15.2 ± 4.2			
Females	0.9 ± 0.3	0.8 ± 0.2	1.0 ± 0.3			

Data are reported as mean ± SD. Significant time, group, and interaction effects are bolded.

#### Plasma volume and associated variables

**Figure 4:**
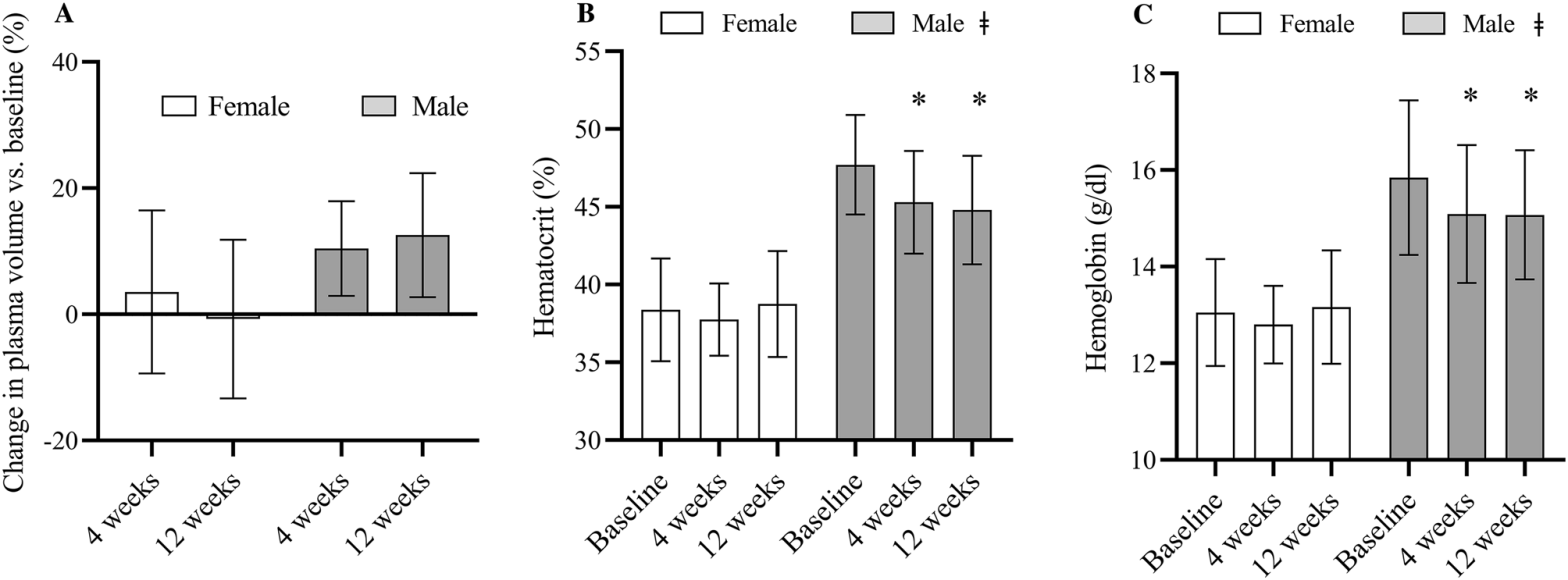
Change in plasma volume (PV; **A**), hematocrit (Hct; **B**) and hemoglobin concentration ([Hb]; **C**) before and after 4 and 12 wk of SIT. Mean (bars) and standard deviation (error bars) are presented. *p < 0.05 between sexes at same time point (ǂ = sex × time interaction).

The mean change in PV after 4 and 12 wk of SIT was 7.4 ± 10.5 and 6.6 ± 12.7%, respectively (Fig. 4a). Hct decreased after SIT (η^2^_p_ = 0.19; main effect of time, *p* = 0.04) and there was a significant sex × time interaction (η^2^_p_ = 0.19, *p* = 0.04; Fig. 4b). Post hoc analysis revealed that Hct was different from baseline (48 ± 5%) after 4 (45 ± 4%, *p* = 0.01) and 12 wk of SIT (45 ± 4%, *p* = 0.003) in the male, but not the female participants (38 ± 3 vs. 38 ± 2 vs. 39 ± 3%, *p* > 0.05 for all). [Hb] also decreased after SIT (η^2^_p_ = 0.22; main effect of time, *p* = 0.02) and there was a sex × time interaction (η^2^_p_ = 0.20, *p* = 0.03; Fig. 4c). Post hoc analysis revealed that [Hb] was different from baseline (16.2 ± 1.6 g/dl) after 4 (15.4 ± 1.4 g/dl, *p* = 0.007) and 12 wk of SIT (15.3 ± 1.3 g/dl, *p* = 0.002) in the male, but not the female participants (13.1 ± 1.1 vs. 12.8 ± 0.8 vs. 13.2 ± 1.2 g/dl, *p* > 0.05 for all).

### Vascular data

#### Resting hemodynamics

Resting HR decreased after SIT (η^2^_p_ = 0.20; main effect of time, *p* = 0.02) and was lower compared to baseline (66 ± 11 bpm) after 4 (62 ± 7; *p* = 0.05) and 12 wk (61 ± 6; *p* = 0.03) following post hoc analysis. Systolic blood pressure (SBP) also decreased after SIT (η^2^_p_ = 0.19, main effect of time, *p* = 0.02) and was lower compared to baseline (115 ± 11 mmHg) after 12 (111 ± 8; *p* = 0.02) but not 4 wk of SIT (112 ± 9; *p* = 0.09). There was a group effect such that males had higher SBP compared to females (117 ± 8 vs. 104 ± 8 mmHg, respectively, η^2^_p_ = 0.72, *p* < 0.001). Diastolic blood pressure (DBP) was unchanged by the intervention (η^2^_p_ = 0.053; *p* = 0.37). There was no sex × time interaction for resting HR, SBP or DBP.

#### Arterial stiffness

There was no effect of time and no sex × time interaction for central or peripheral leg or peripheral arm PWV (*p* > 0.05 for all; Table 3). Carotid artery distensibility increased after SIT (η^2^_p_ = 0.17; main effect of time, *p* = 0.04) and was higher compared to baseline (0.005 ± 0.001 mmHg^−1^) after 12 (0.006 ± 0.002; *p* = 0.04) but not 4 wk of SIT (0.006 ± 0.002; *p* = 0.15) following post hoc analyses. There was no effect of time (η^2^_p_ = 0.091, *p* = 0.18) and no sex × time interaction (η^2^_p_ = 0.072, *p* = 0.26) for carotid artery compliance. There was a group effect such that males had higher compliance than females (0.185 ± 0.05 vs. 0.135 ± 0.03 cm^2^/mmHg, respectively, η^2^_p_ = 0.55, *p* = 0.003). There was a sex × time interaction for β-stiffness (η^2^_p_ = 0.20, *p* = 0.02) with post hoc analyses revealing that female participants had a higher β-stiffness than male participants at wk 12 (5.6 ± 0.7 vs. 4.3 ± 0.9 a.u., respectively, *p* = 0.003).

**Table 3:** Carotid arterial stiffness.

	Week 0	Week 4	Week 12	Time effect	Group effect	Sex × time interaction
Arterial stiffness						
Central PWV (m/s)						
Overall	6.1 ± 2	5.8 ± 1	6.5 ± 2	η^2^_p_ = 0.063 *p* = 0.31	η^2^_p_ = 0.075 *p* = 0.35	η^2^_p_ = 0.082 *p* = 0.21
Males	6.3 ± 2	6.5 ± 2	6.5 ± 2			
Females	6.0 ± 2	5.2 ± 1	6.4 ± 2			
Peripheral leg PWV (m/s)						
Overall	8.5 ± 2	8.1 ± 2	7.4 ± 1	η^2^_p_ = 0.14 *p* = 0.07	η^2^_p_ = 0.012 *p* = 0.61	η^2^_p_ = 0.009 *p* = 0.86
Males	8.7 ± 1	8.1 ± 2	7.6 ± 1			
Females	8.3 ± 2	8.1 ± 2	7.3 ± 1			
Peripheral arm PWV (m/s)						
Overall	5.9 ± 2	5.9 ± 2	6.7 ± 2	η^2^_p_ = 0.10 *p* = 0.15	η^2^_p_ = 0.021 *p* = 0.64	η^2^_p_ = 0.056 *p* = 0.36
Males	5.9 ± 2	6.5 ± 2	6.7 ± 3			
Females	6.0 ± 2	5.4 ± 1	6.7 ± 2			
Carotid artery stiffness						
Compliance (cm^2^ × mmHg ^−1^)						
Overall	0.15 ± 0.04	0.16 ± 0.05	0.17 ± 0.06	η^2^_p_ = 0.091 *p* = 0.18	η^2^_p_ = 0.55 ***p* = 0.003**	η^2^_p_ = 0.072 *p* = 0.26
Males	0.17 ± 0.03	0.18 ± 0.05	0.20 ± 0.06			
Females	0.13 ± 0.04	0.14 ± 0.03	0.37 ± 0.04			
Distensibility (mmHg^−1^)						
Overall	0.005 ± 0.001	0.006 ± 0.002	0.006 ± 0.002*	η^2^_p_ = 0.17 ***p* = 0.04**	η^2^_p_ = 0.32 *p* = 0.06	η^2^_p_ = 0.080 *p* = 0.22
Males	0.006 ± 0.001	0.007 ± 0.002	0.007 ± 0.003			
Females	0.005 ± 0.001	0.005 ± 0.001	0.005 ± 0.000			
β-stiffness (a.u.)						
Overall	4.88 ± 0.72	4.84 ± 1.01	4.96 ± 1.1	η^2^_p_ = 0.011 *p* = 0.82	η^2^_p_ = 0.29 ***p* = 0.05**	η^2^_p_ = 0.20 ***p* = 0.02**
Males	4.76 ± 0.54	4.66 ± 1.10	4.29 ± 0.98			
Females	4.99 ± 0.87	5.01 ± 0.94	5.64 ± 0.67			
Carotid intima media thickness (mm)						
Overall	0.37 ± 0.04	0.37 ± 0.03	0.37 ± 0.04	η^2^_p_ = 0.002 *p* = 0.97	η^2^_p_ = 0.078 *p* = 0.53	η^2^_p_ = 0.028 *p* = 0.60
Males	0.36 ± 0.03	0.37 ± 0.03	0.36 ± 0.03			
Females	0.37 ± 0.04	0.37 ± 0.04	0.38 ± 0.05			

Data are reported as mean ± SD. Significant time, group, and interaction effects are bolded.

PWV, Pulse wave velocity.

*p < 0.05 for post hoc testing between time points compared to baseline.

#### Endothelial function

There was a group effect such that males had larger baseline (η^2^_p_ = 0.99, *p* < 0.001) and peak (η^2^_p_ = 0.98, *p* < 0.001) brachial arterial diameters compared to females (Table 4). There was a main effect of sex such that %FMD was greater in females compared to males, however after allometrically scaling (4) to control for the differences in baseline diameter, apparent sex differences in %FMD were no longer present (Males: 7.5 ± 3.2%; Females: 7.6 ± 3.2%). There was no effect of time on %FMD, unscaled or scaled. There was no effect of time and no sex × time interaction for mean blood velocity, shear rate, or %FMD / shear rate area under the curve of deflation to peak dilation (*p* > 0.05 for all; Table 4. There was a sex × time interaction for time to deflation (η^2^_p_ = 0.16, *p* = 0.05), though there was no effect of time (*p* = 0.48).

**Table 4:** Endothelial function and blood flow measures.

	Week 0	Week 4	Week 12	Time effect	Group effect	Sex × time interaction
Endothelial function						
Brachial artery baseline diameter (mm)						
Overall	3.8 ± 0.7	3.8 ± 0.7	3.8 ± 0.7	η^2^_p_ = 0.012 *p* = 0.80	η^2^_p_ = 0.99 ***p* < 0.001**	η^2^_p_ = 0.083 *p* = 0.21
Males	4.3 ± 0.5	4.3 ± 0.5	4.3 ± 0.5			
Females	3.2 ± 0.4	3.2 ± 0.4	3.2 ± 0.4			
Brachial artery peak diameter (mm)						
Overall	4.0 ± 0.7	4.0 ± 0.7	4.0 ± 0.7	η^2^_p_ = 0.039 *p* = 0.49	η^2^_p_ = 0.98 ***p* < 0.001**	η^2^_p_ = 0.028 *p* = 0.60
Males	4.5 ± 0.5	4.6 ± 0.5	4.6 ± 0.6			
Females	3.5 ± 0.4	3.5 ± 0.3	3.5 ± 0.4			
Absolute FMD (mm)						
Overall	0.26 ± 0.1	0.28 ± 0.1	0.27 ± 0.1	η^2^_p_ = 0.030 *p* = 0.58	η^2^_p_ = 0.11 *p* = 0.48	η^2^_p_ = 0.033 *p* = 0.55
Males	0.26 ± 0.2	0.26 ± 0.1	0.25 ± 0.1			
Females	0.26 ± 0.1	0.30 ± 0.1	0.30 ± 0.1			
Relative FMD (%)—Unscaled						
Overall	7.2 ± 3	7.9 ± 4	7.6 ± 4	η^2^_p_ = 0.038 *p* = 0.49	η^2^_p_ = 0.55 ***p* = 0.03**	η^2^_p_ = 0.052 *p* = 0.38
Males	6.0 ± 3	6.0 ± 3	5.7 ± 3			
Females	8.3 ± 3	9.7 ± 5	9.5 ± 3			
Relative FMD (%)—Allometrically scaled						
Males	7.5 ± 1	7.6 ± 1	7.3 ± 1	*p* = 0.46	*p* = 0.96	*p* = 0.46
Females	6.7 ± 1	8.0 ± 1	7.3 ± 1			
Blood flow						
Brachial artery baseline mean blood velocity (cm/s)						
Overall	7.2 ± 4	8.2 ± 4	7.6 ± 3	η^2^_p_ = 0.050 *p* = 0.38	η^2^_p_ = 0.27 *p* = 0.10	η^2^_p_ = 0.10 *p* = 0.14
Males	8.6 ± 4	9.7 ± 5	7.9 ± 3			
Females	5.8 ± 2	6.6 ± 2	7.4 ± 2			
Brachial artery baseline shear rate (s^−1^)						
Overall	152 ± 67	177 ± 77	169 ± 67	η^2^_p_ = 0.075 *p* = 0.25	η^2^_p_ = 0.002 *p* = 0.91	η^2^_p_ = 0.12 *p* = 0.11
Males	165 ± 82	186 ± 101	152 ± 77			
Females	140 ± 49	168 ± 44	186 ± 55			
%FMD / shear rate area under the curve of deflation to peak dilation						
Overall	0.39 ± 0.3	0.34 ± 0.2	0.34 ± 0.3	η^2^_p_ = 0.032 *p* = 0.55	η^2^_p_ = 0.15 *p* = 0.26	η^2^_p_ = 0.014 *p* = 0.78
Males	0.32 ± 0.3	0.30 ± 0.2	0.29 ± 0.2			
Females	0.48 ± 0.3	0.39 ± 0.2	0.39 ± 0.3			
Time to deflation (s)						
Overall	53 ± 26	48 ± 13	53 ± 19	η^2^_p_ = 0.040 *p* = 0.48	η^2^_p_ = 0.064 *p* = 0.40	η^2^_p_ = 0.16 ***p* = 0.05**
Males	63 ± 32	48 ± 14	53 ± 23			
Females	43 ± 12	49 ± 13	54 ± 15			

Data are reported as mean ± SD. Significant time, group, and interaction effects are bolded.

FMD, flow mediated dilation.

* *p* < 0.05 for post hoc testing between time points compared to baseline.

## Discussion

The primary finding of this study was that biological sex did not affect the Q̇_peak_ response to 12 wk of SIT in young healthy adults who were previously untrained. While SIT increased V̇O_2peak_, Q̇_peak_ was unchanged. This finding contrasts with our hypothesis and a previous study from our laboratory with a similar study design that reported a 12-wk SIT intervention increased both V̇O_2peak_ and Q̇_peak_^11^. The reason for the discrepant results between studies is unclear but may relate to interindividual differences in training responsiveness that are not explicitly linked to biological sex. These data also collectively suggest that increased skeletal muscle oxygen extraction or diffusing capacity are an important contributor to SIT-induced increases in V̇O_2peak_. The present study also found minimal impact of SIT on vascular outcomes, including arterial stiffness and endothelial function. Notably, SIT was associated with decreased resting HR and SBP in both males and females and carotid artery β-stiffness in males.

SIT increased V̇O_2peak_ by 10% in the present study, which is comparable to previous work^7,9,10,17,18,32–34^ and within the range of 2–12 ml/kg/min that is typical of interval training interventions lasting 4–12 wk^35^. The effect size for the change in V̇O_2peak_ over 12 wk was large (> 0.14)^36^ in both the present study (η^2^_p_ = 0.53) and our previous study (η^2^_p_ = 0.58)^11^, although the magnitude of change was lower in the present study (~ 10 vs. 20%). V̇O_2peak_ measured using the Innocor during the Q̇_peak_ tests also increased following SIT, but in accordance with our previous work^37^, the values were consistently ~ 25% lower than those measured using the Quark CPET metabolic cart^37^. Contrary to our previous study^11^, although in line with some other studies^16,33,34,38^, there was no sex-based difference in the change in V̇O_2peak_ following SIT. Given the state of the literature and that the current study was specifically designed to make sex-based comparisons, we conclude that there is no sex-based difference in V̇O_2peak_ responses to SIT.

Our finding that 12 wk of SIT did not alter Q̇_peak_ contrasts with our previous study that reported an increase in Q̇_peak_ after a similar training intervention^11^. Data regarding the effect of SIT on Q̇_peak_ remains limited and equivocal. The time course for changes as well as inter-individual differences in training responsiveness that may or may not be influenced by biological sex are not well understood. In addition to our previous study^11^, Mandić et al.^10^ also reported an increase in non-invasively determined Q̇_peak_ after 6 wk of SIT, and this was confirmed in another recent study by the same group that employed the gold-standard direct Fick method^18^. In contrast, the present study and previous literature have reported no change in Q̇_peak_ despite a ≥ 10% increase in V̇O_2peak_^7,9^. In the recent studies by Mandić et al. (10,18), the SIT protocol consisted of 3 × 30-s ‘all-out’ sprints separated by 2 min of recovery, which differs from the 20-s sprints used in the present study. Aerobic metabolism constitutes ~ 50% of the energy provision in the last 15 s of a 30-s ‘all-out’ sprint^39,40^. Therefore, the extra 10 s of sprinting used in the Mandic studies likely places greater cumulative stress on the oxygen delivery system which may elicit more pronounced cardiovascular responses and lead to an increase in Q̇_peak_. The training data from Mandic et al.^10^ revealed that the sprints elicited a mean V̇O_2_ that was 98% of V̇O_2peak_. This likely coincides with close to 100% HR_peak_^41^, which was greater than the mean HR achieved during the sprints of our study, which was 92% HR_peak_.

We were unable to replicate the increase in Q̇_peak_ from our previous study despite similar participant characteristics and a similar SIT protocol that elicited a near identical stimulus. Mean HR during the 20-s sprints was 92% of HR_peak_, and mean HR over the 10-min session was 80% of HR_peak_ in both studies^11^. The reason for the discrepant findings between the two studies from our laboratory is unclear but it may be related to inter-individual differences in training responsiveness. The potential reasons for such differences are multifaceted. Recent reviews have considered directions for examining the issue of exercise treatment response heterogeneity and approaches to classify individual responses to exercise training^42,43^. There is considerable variability in the trainability of V̇O_2peak_ following exercise training^44–47^, which likely extends to Q̇_peak_. Large variability has also been seen in the V̇O_2peak_ response to SIT protocols^34^. Figure 2 demonstrates that the 6 male participants in our previous study were relatively large “responders” to the SIT intervention. Aside from these individuals, the overall pattern of response for the female participants was similar between the two studies with relatively little change in Q̇_peak_ that was generally within the margin of error.

The lack of change in Q̇_peak_ observed in the current study suggests the improvement in V̇O_2peak_ was mainly attributable to an enhanced peak a-vO_2diff_, based on the Fick principle^6^. Increases in peak a-vO_2diff_ that contribute to the SIT-induced improvement in V̇O_2peak_ is in agreement with our previous study where improvements in Q̇_peak_ and peak a-vO_2diff_ were both associated with the increase in V̇O_2peak_^11^. Similarly, the study by Mandic et al.^18^ found an increase in directly measured Q̇_peak_ and systemic oxygen extraction following SIT. This finding is corroborated by meta-analyses that report shorter (≤ 30-s) intervals improve V̇O_2peak_ primarily through peripheral adaptations related to skeletal muscle oxygen extraction^44,48^. In addition to training studies, the mechanistic role that peak a-vO_2diff_ plays in the regulation of V̇O_2peak_ is demonstrated in studies that experimentally alter oxygen delivery^18,49–51^. One set of experiments found that in untrained individuals, the maximal in vivo whole-body delivery of oxygen to skeletal muscle was greater than in vitro oxygen consumption in maximally perfused skeletal muscle samples, suggesting oxygen extraction was the limiting factor to oxygen consumption^50^. Studies that use phlebotomy to experimentally alter Q̇_peak_ have shown mixed results. V̇O_2peak_ has been found to remain above baseline levels when post training increases in blood volume and Hb_mass_ were reverted to baseline through phlebotomy, which suggests peripheral adaptations contributed to the training-induced improvement in V̇O_2peak_^49^. Conversely, other studies using a similar experimental design found complete restoration of V̇O_2peak_ back to baseline levels following phlebotomy^52,53^. One study using SIT found that when increases in blood volume and Q̇_peak_ were reverted to baseline using phlebotomy, the change in V̇O_2peak_ appeared to remain elevated despite not being significantly different from baseline (*p* = 0.06)^18^. Esposito et al.^51^ found that 8 wk of endurance training using a kicking exercise model resulted in improved V̇O_2peak_ and peripheral muscle adaptations including capillarization and enhanced muscle oxygen diffusing capacity^51^. These adaptations occurred in the absence of an improvement in Q̇_peak_, suggesting peripheral responses drove the increase in V̇O_2peak_. SIT increases mitochondrial content and capillary density^54–56^, which could facilitate the improvement in peak a-vO_2diff_ following SIT. Improved skeletal muscle oxygen diffusing capacity can increase peak a-vO_2diff_^51^, but whether it is affected by SIT is unknown.

PV expansion in our study was similar in magnitude to two recent studies showing that SIT increased PV by 6–8% measured using the gold-standard carbon monoxide rebreathing technique^10,18^. PV was estimated in our study using the Dill and Costill method^57^, which calculates a percent change from baseline using changes in Hct and [Hb]. We therefore did not run statistics on the change in PV; however, there was a significant change in Hct and [Hb] following SIT. In addition, we found a significant sex × time interaction for changes in Hct and [Hb], with post hoc analyses revealing decreases in the male, but not the female participants, following SIT. This suggests a potential sex-based difference in PV expansion following SIT, which has not previously been reported in the literature. Sex-based differences in post-training PV expansion, regardless of training type, have not been investigated previously. The mechanistic underpinnings for the lack of response among the female participants may be related to a recent finding that females accrue a smaller build-up of exercise-induced metabolites following an acute bout of SIT compared to males^28,58^, which could be due to the preferential oxidation of fat vs. carbohydrates in females^59^. Recent work suggests that the accumulation of these metabolites, particularly glucose-6-phosphate, may be a primary mechanism in SIT induced PV expansion^60^. Therefore, it is possible that while the relative HR during SIT was greater among female participants in the present study, the metabolic stress may have been lower, which could have resulted in a blunted signalling response for the increase in PV. Given our study was not powered to detect a sex-based difference in PV and used an indirect method to estimate change in PV, future studies should further explore the possibility of a sex-based difference in PV following SIT using gold-standard measures and assess the associated mechanisms. PV, and subsequent red blood cell volume expansion, is a mechanism behind exercise-induced increases in Q̇_peak_ because of the Frank Starling mechanism^6^. The lack of increase in Q̇_peak_ in the current study despite the presence of PV expansion could be due to the non-invasive nature of the IGR procedure, which may have lacked the sensitivity to detect subtle changes in Q̇_peak_. In our previous study, the magnitude of the SIT-induced increase in Q̇_peak_ was relatively small (+ 1.1 L/min, post hoc 12 wk vs. baseline *p* = 0.05). As previously noted, the overall responsiveness of the participants to the intervention in the present study was smaller than our previous study^11^, despite an identical exercise stimulus. It is possible that this lack of notable response is due to the large inter-individual variability in physiological responses to exercise training^44,45^. It is possible that a small change in Q̇_peak_ went undetected in the current study. The change in PV was not associated with the change in V̇O_2peak_ (r^2^ = 0.006, *p* = 0.76) possibly because of the corresponding decrease in oxygen carrying capacity, as demonstrated by the reduction in Hct and [Hb] in the present study.

This study considered the influence of SIT on resting hemodynamics, arterial stiffness, and endothelial function, as factors influencing blood flow delivery to working skeletal muscle. The present study found that 4 and 12 wk of SIT had no effect on arterial stiffness or endothelial function, and no sex-differences were present in these outcomes. The arterial stiffness findings are consistent with previous literature^27,29,61^, including work from our laboratory by Shenouda and colleagues^29^ that showed central and leg PWV did not change with 6 or 12 wk of SIT in male participants. The present study may have been underpowered to detect a difference, as a reduction in the arterial stiffness of the exercising was previously reported after 6 wk of SIT, although the sprints in that study were 30 s in duration^27^. Although non-significant, there was an observed decrease in leg PWV of ~ 1 m/s, which may be clinically relevant as a 1 m/s decrease in central arterial stiffness is associated with a 15% decreased risk of having a cardiovascular event^62^. In contrast, SIT did not impact brachial artery endothelial function, which is concurrent with findings previously reported by our lab at 6 and 12 wk of SIT in males^29^. There is previous literature to suggest that 6 wk of SIT improves endothelial function in the popliteal artery, however, endothelial function of the exercising limb was not measured in the present study^27^. It is possible that SIT may improve endothelial function in the exercising vascular bed (i.e., legs) but not in the inactive limb (i.e., arms), but further research is needed to confirm this hypothesis.

Despite the lack of findings in peripheral arterial stiffness and endothelial function, there was some evidence of improvements in central cardiovascular outcomes with SIT. This study found that carotid artery distensibility improved with SIT, which contrasts with one previous study observing no change in distensibility with SIT^27^. However, this may be attributed to the difference in protocol duration (i.e., 6 vs. 12 wk). It is expected that adaptations in arterial stiffness may occur later in a training protocol compared to functional changes which manifest earlier in the time course^30^. Although females had superior carotid artery compliance compared to males, β-stiffness was improved in males compared to females at 12 wk of SIT. The mechanisms underlying the improved β-stiffness in males seen in this study are unclear. Contrary to our results, previous studies have reported that β-stiffness is elevated in males at baseline compared to females^63^, and that elevated β-stiffness at baseline corresponds with greater reductions in stiffness with high intensity aerobic training^27^. The mechanism underlying elevated β-stiffness in males may be higher systolic blood pressure; a ~ 14 mmHg difference between males and females was seen in the present study at baseline. This elevated blood pressure may have predisposed males to greater carotid artery adaptations compared to females through structural adaptations to align with a decrease in blood pressure after SIT (~ 6 mmHg decrease wk 0–12).

A limitation of the IGR procedure is that it may underestimate Q̇_peak_ due to nitrous oxide recirculation^64^. The underestimation of Q̇_peak_ likely inflated our calculation of peak a-vO_2diff_, which was estimated using the Fick equation (i.e., peak a-vO_2diff_ = V̇O_2peak_ / Q̇_peak_). Notwithstanding this limitation, the IGR procedure is highly correlated with gold standard methods^65^ and we recently demonstrated that the day-to-day repeatability of Q̇_peak_ using IGR was similar to that of V̇O_2peak_^37^. A strength of our study was the controls used for making sex-based comparisons. We attempted to match the two groups of participants for V̇O_2peak_ relative to FFM, as the approach is deemed best practice for making sex-based comparisons of exercise responsiveness^66^. Participants also performed the same number of training sessions. The female participants completed all testing sessions in the low hormone phase of their cycle, which was confirmed by venous blood hormone analysis. This is currently considered best-practice because of the potential effect of estradiol and progesterone on HR and V̇O_2_^12^. There is no explicit estradiol or progesterone cut-off that constitutes the ‘low hormone phase’ because of the naturally large variability in hormonal profiles between female participants^67^. However, the estradiol and progesterone levels measured in the female participants in our study (138 ± 52 pmol/L and 3.6 ± 1.5 nmol/L, respectively) are similar to those reported in the literature for the early follicular phase (i.e., ~ 120 pmol/L and < 5 nmol/L, respectively)^12,68^ and were not different from the male participants.

In conclusion, the present study found no change in Q̇_peak_ following 12 wk of SIT despite an increased V̇O_2peak_, and no sex-based differences in either measure. Our results suggest that an increased capacity for muscle oxygen extraction or diffusing capacity plays an important role in the SIT-induced increase in V̇O_2peak_ SIT. The capacity of the non-invasive IGR method to robustly assess changes in Q̇_peak_ may be limited and invasive studies that use more direct measures are needed to clarify the precise time course and physiological determinants of the increase in V̇O_2peak_. Twelve wk of SIT also had relatively little impact on measures of arterial stiffness and endothelial function. Resting heart rate and systolic blood pressure was lower after training in both sexes, and carotid artery stiffness was reduced in males. The mechanistic basis for these changes remains to be determined but is suggestive of improved resting cardiovascular function.

## Methods

### Participants

An a-priori sample size calculation (G*Power v 3.1.9.2), based on data from our previous study that detected an exploratory sex-based difference in Q̇_peak_^11^, estimated that 14 participants were required to detect a partial eta-squared (η^2^_p_) of 0.46 with 80% power at α = 0.05 for a 2 × 2 (time × group) mixed analysis of variance (ANOVA) with 2 time points (baseline vs. follow-up) and 2 groups (male vs. female). To preserve power, we sought to recruit 20 participants with 10 males and 10 females in each group. A total of 11 females were recruited; one participant withdrew during the study after a change in their health status made them ineligible to continue. The final group characteristics are summarized in Table 1. Among the female participants, 6 were eumenorrheic naturally menstruating and 4 were using a monophasic 2nd generation oral contraceptive (Alesse/Alysena: 20 mcg ethinyl estradiol and 100 mcg levonorgestrel 1/day for 21 days). Female participants were tested in the low hormone phase of their menstrual cycle (i.e., early follicular phase; days 2–7 of cycle) or oral contraceptive pill phase (i.e., placebo/hormone withdrawal phase). Participants were deemed untrained based on a self-report of engaging in < 1 h of weekly moderate to vigorous physical activity based on the Canadian Society for Exercise Physiology Get Active Questionnaire^69^. This study was approved by the Hamilton Integrated Research Ethics Board (Project # 14279) and all methods were performed in accordance with the relevant guidelines and regulations. All participants provided written informed consent prior to participation. The study was registered on ClinicalTrials.org prior to participant recruitment (NCT05205538) on 25/01/2022.

### Overview of experimental procedures

#### Familiarization and baseline testing

**Figure 5:**
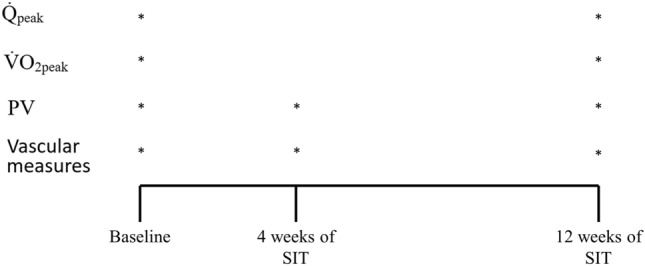
Study overview.

An overview of the study design is presented in Fig. 5. This study used a 2-factor repeated measures, mixed design (between factor: sex, within factor: time) to compare changes between males and females in response to 12 wk of SIT. All data collection took place in the Human Performance Laboratory and the Vascular Dynamics Laboratory at McMaster University in Hamilton, Ontario, Canada. Following preliminary screening and recruitment into the study, participants completed a single-question questionnaire to determine their biological sex. Before baseline testing, participants attended the laboratory to perform a familiarization session with the cardiac output IGR and the FMD procedures. First, participants sat in an upright position with a blood pressure cuff placed on the distal forearm, which was subsequently inflated to 200 mmHg for 5 min to simulate the artery occlusion portion of the FMD procedure. Participants then cycled at a self-selected moderate intensity for 5 min, at which point the IGR procedure was initiated. Baseline testing consisted of 2 visits to the laboratories on back-to-back days. Participants arrived after a 10 h fast and abstained from any structured exercise and alcohol consumption for a minimum of 24 h before testing. Upon arrival to the laboratory, body composition was measured using air displacement plethysmography to determine FFM (BodPod, COSMED, Italy). Participants then underwent vascular testing. After 10 min of supine rest, 8 mL of venous blood was drawn from an antecubital vein. Four mL of blood was immediately analyzed for Hct and [Hb] using a handheld blood gas analyzer (epoc Blood Analysis System, Siemens, Munich, Germany) to estimate PV. The remaining 4 mL of blood was used for the subsequent analysis of estradiol, progesterone, and testosterone concentrations. Following the blood draw, participants were instrumented non-invasively for the measurement of continuous heart rate (HR) and blood pressure using a single lead ECG (model ML 132; ADInstruments, Colorado Springs, CO) and photoplethysmographic blood pressure unit (Finometer MIDI, Finapres Medical Systems; Amsterdam, The Netherlands), respectively. Participants then underwent testing for arterial stiffness using applanation tonometry (Mikro-Tip Catheter Tranducer, model CPT-301; Millar Instruments) to determine central and peripheral PWV. Arterial stiffness was also assessed at the carotid artery using simultaneous applanation tonometry and Doppler ultrasound imaging (Vivid Q; GE Medical Systems, Horten Norway). This was followed by an FMD test of the brachial artery, using Doppler ultrasound, to measure endothelial function. Participants returned to the laboratory the next day to perform a V̇O_2peak_ test (Quark CPET metabolic cart, COSMED, Italy) and a Q̇_peak_ test (Innocor, COSMED, Italy).

Training began ~ 48 h following the last baseline test and lasted for 12 wk, with 3 supervised sessions performed each wk. Participants performed their 4-wk follow-up assessments in place of the first SIT session of the 5th wk of training. This involved a single visit to the laboratory and included FMD and arterial stiffness (PWV, distensibility) assessments and an 8 mL venous blood draw to assess PV and estradiol, progesterone, and testosterone content. The 12-wk measurements commenced ~ 48 h after the last training session. These sessions were identical to the baseline tests except body composition was not measured. Venous blood data for hormone analyses was available for 19/20 participants due to inability to draw blood from one participant, and blood data for the change in PV calculation is available for 18/20 participants due to technical difficulties with the blood analysis for one additional participant.

#### Training intervention

The training intervention was modelled on previous studies from our laboratory^11,70^ and involved 3 weekly SIT sessions for 12 wk on a cycle ergometer (CAROL Bike, Integrated Health Partners, London, UK). Each session involved a 2-min warm-up, 3 × 20-s ‘all-out’ sprints against an individualized resistance determined by a self-learning algorithm that adjusts based on the participant’s weight, power output, and fatigue index, interspersed with 2 min of recovery, and a 3-min cool-down. Training was performed with ≥ 24 h between sessions. HR was measured continuously during training sessions (Polar A3, Kempele, Finland). Aside from the supervised exercise intervention, participants were instructed to make no changes to their habitual lifestyle, including physical activity and diet, for the duration of the study period.

### Measurements

#### Peak oxygen uptake

Participants performed a progressive exercise test to maximal voluntary exertion using an electromagnetically braked cycle ergometer (Lode Excalibur Sport V2.0, Groningen, The Netherlands). Following a 3 min warm-up at a fixed workload of 50 W, a ramp protocol was applied with a linear workload increase of 1 W every 2 s (30 W/min). Pedaling cadence was chosen by the participant and was required to be ≥ 60 rpm. Failure to maintain a cadence ≥ 60 rpm was considered voluntary exhaustion and the test was stopped. A 3-min recovery phase was performed at 50 W. Gas exchange and ventilatory variables were continuously determined using a metabolic cart (Quark CPET, COSMED, Italy). These data were averaged over 10-s intervals and V̇O_2peak_ was defined as the highest 30-s average over three consecutive intervals. HR was recorded continuously (Polar A3, Kempele, Finland) and HR_peak_ was defined as the highest 1-s peak. Data-based cut-offs for age-stratified secondary exhaustion criteria based on peak respiratory exchange ratio (RER ≥ 1.13) and age-predicted maximal HR (Eq. 1)^71^ were used to verify that the test involved maximal effort.

1 $$0.{93}*\left( {{2}0{8} - \left( {0.{7}*{\text{age}}} \right)} \right)$$

#### Peak cardiac output

Q̇_peak_ was assessed non-invasively using IGR (Innocor, COSMED, Italy) as previously described^37^. The IGR procedure involved taking 5–6 breaths from a closed circuit rebreathing bag containing a mixture of oxygen (95%), an inert blood soluble gas (nitrous oxide, 5%), and an inert blood insoluble gas (sulfur hexafluoride, 1%)^72^. Photoacoustic gas analyzers monitored the expired air and measured the disappearance rate of the blood soluble gas relative to the blood insoluble gas over the course of the rebreathing period to estimate Q̇.

The exercise protocol for assessing Q̇_peak_ was modelled after V̇O_2peak_ tests that involve a post-test constant load high-intensity exercise bout designed to re-elicit V̇O_2peak_. Studies show that constant load exercise performed at > 85% W_peak_ can elicit comparable V̇O_2_ values, and HR values that are within ~ 5% of those obtained at the end of a ramp V̇O_2peak_ test^9,11,37,73,74^. The protocol was performed on a cycle ergometer (Lode Excalibur Sport V2.0, Groningen, The Netherlands) and took place 10 min after performing the V̇O_2peak_ test. The protocol involved a 2-min warm-up at 50 W, followed by an immediate increase to an intensity equivalent to 90% of the W_peak_ elicited during the V̇O_2peak_ test (90% W_peak_). The IGR procedure was initiated after 2 min of cycling at 90% W_peak_. The volume of the rebreathing bag was automatically customized to each participant using the tidal volume measured during the lead-up to the rebreathing. A recent study from our laboratory demonstrated that this Q̇_peak_ exercise protocol elicits Q̇_peak_ values that are similar to other routinely used exercise tests for assessing Q̇_peak_, has comparable day-to-day repeatability to V̇O_2peak_ tests (typical error = 6.6%; intraclass correlation coefficient = 0.94), and elicits V̇O_2_ responses similar to the values elicited during a V̇O_2peak_ test^37^. HR (Polar A3, Kempele, Finland) and V̇O_2_ (Innocor, COSMED, Italy) were monitored continuously throughout the test.

Peak a-vO_2diff_ was calculated as V̇O_2peak_ / Q̇_peak_ based on the 2 separate tests performed at a given time point. Peak cardiac index (CI_peak_) is reported with Q̇_peak_ and calculated as Q̇_peak_ / body surface area. Body surface area was calculated using the Mosteller formula (Eq. 2)^75^:

2 $$0.0{16667 } \times \, \left( {\text{body mass}} \right)^{{0.{5}}} \times \, \left( {{\text{height}}} \right)^{{0.{5}}}$$

#### Sex hormone analyses

Sex hormone analyses were conducted to confirm that female participants were tested in their low hormone phase and to compare hormone levels between males and females. A 4 mL tube of blood was collected from an antecubital vein for serum analysis of 17β-estradiol, progesterone, and testosterone concentrations. Blood was stored at room temperature to coagulate for 45 min, followed by rapid centrifugation at 4000 rpm for 10 min at 4 °C. Serum was aliquoted into 1 mL storage vials and frozen at − 80 °C for later batch analysis. Hormone levels were analyzed batched by participant for baseline, 4 and 12 wks in triplicate at the Hamilton Regional Medicine Program Core Laboratory for analysis to analyze for serum concentrations of 17β-estradiol (Architect Estradiol Chemiluminescent Microparticle Immunoassay, sensitivity < 92 pmol/L, Abbott Diagnostics, Abbott Park, IL), progesterone (Architect Progesterone Chemiluminescent Microparticle Immunoassay, sensitivity < 0.3 nmol/L, Abbott Diagnostics), and testosterone (Immulite 2000 chemiluminescent enzyme immunoassay, sensitivity < 0.7 nmol/L, Siemens Healthcare Diagnostics, Tarrytown, NY). Any values beneath detectable limits were assumed to be the lowest detectable value for inclusion in the analysis.

#### Plasma volume

Following 10 min of supine rest, 4 mL of blood was drawn from an antecubital vein into a vacutainer tube containing ethylenediamine tetraacetic acid (EDTA) and analyzed immediately for [Hb] and Hct using a handheld blood gas analyzer (epoc Blood Analysis System, Siemens, Munich, Germany). The change in PV from pre-training to each of the 4-wk and 12-wk time points was estimated using the following formula per the Dill and Costill method (Eq. 3)^57^:

3 $$\Delta {\text{PV }}\left( \% \right) \, = \, \left( {\left( {\left( {\left[ {{\text{Hb}}} \right]_{{{\text{pre}}}} \times \, \left( {{1 }{-}{\text{ hematocrit}}_{{{\text{post}}}} } \right)} \right) \, / \, \left( {\left[ {{\text{Hb}}} \right]_{{{\text{post}}}} \times \, \left( {{1 }{-}{\text{ hematocrit}}_{{{\text{pre}}}} } \right)} \right)} \right) \, {-}{ 1}} \right) \, \times { 1}00$$

#### Vascular outcomes

FMD was measured from the left brachial artery using Doppler ultrasound technology (Vivid Q; GE Medical Systems, Horten Norway). Briefly, a baseline ultrasound image and blood flow in the artery was acquired for 30 s, followed by rapid inflation of a blood pressure cuff positioned at the distal forearm to suprasystolic pressure (200 mmHg) using a rapid inflator, for 5 min (E20 Rapid Cuff Inflator and AG101 Air Source; Hokanson, Bellevue, WA). Following cuff deflation, 3 min of imaging and blood flow was acquired. Ultrasound images were saved offline as Digital Imaging and Communications in Medicine (DICOM) files and were processed through a software that allowed for the capture of end-diastolic values of the arterial diameter (Sante DICOM Editor, v. 3.1.20, Santesoft, Athens, Greece). Images were blinded and analyzed by a single investigator (J.S.W.) and analyzed using semiautomated edge tracking software (Artery Measurement System AMS) II version 1.141; Gothenburg, Sweden) to determine the baseline and peak arterial diameters. Absolute FMD was calculated as the difference between peak and baseline artery diameter. %FMD was calculated as absolute FMD/baseline diameter × 100%. Blood velocity traces were saved offline as AVI files and were analyzed using a pixel-based software (Measurements from Arterial Ultrasound Imaging; Hedgehog Medical, Waterloo, ON, Canada).

PWV was assessed at the left carotid and femoral arteries (central PWV), carotid and radial arteries (peripheral arm PWV), and femoral and dorsalis pedis arteries (peripheral leg PWV), to fully characterize stiffness alternations with training. In rare instances in which the dorsalis pedis artery was not easily located, the posterior tibial artery was used for all testing sessions (n = 2). An applanation tonometer (Mikro-Tip Catheter Tranducer, model CPT-301; Millar Instruments) was placed at each pulse point of interest on the left side of the body until pulse waveforms of sufficient quality are obtained for ~ 30 heart cycles. The pulse transit time was determined using digitally filtered (band pass, 5–30 Hz) pressure waveforms, simultaneously detected at each combination of artery sites using commercially available software (Powerlab model ML870, ADInstruments). Following acquisition of the signals, an anthropometric tape measure was used to determine distances across the artery sites for later analysis. PWV was analyzed using analysis software (LabChart 8, ADInstruments) as the mean of 2 sets of 10 heart cycles. PWV was calculated using Eqs. (4)–(6):

4 $${\text{Central PWV }} = \left( {0.{8}*{\text{carotid-femoral distance}}} \right)/{\text{carotid-femoral pulse transit time}})$$

5 $${\text{Peripheral arm PWV }} = \left( {\left( {{\text{radial-suprasternal notch distance }}{-}\left( {{\text{carotid}}{-}{\text{suprasternal notch distance}}} \right)} \right)/{\text{carotid}}{-}{\text{radial pulse transit time}}} \right)$$

6 $${\text{Peripheral leg PWV }} = \, \left( {{\text{femoral}}{-}{\text{dorsalis pedis distance}}} \right)/{\text{femoral}}{-}{\text{dorsalis pedis transit time}})$$

Carotid artery distensibility, compliance, and β-stiffness of the common carotid artery (CCA) were assessed using a combination of 12 Hz Doppler ultrasound imaging on the left CCA (Vivid Q; GE Medical Systems, Horten, Norway) and applanation tonometry on the right CCA (Mikro-Tip Catheter Tranducer, model CPT-301; Millar Instruments). Ten consecutive and simultaneous average arterial diameters and pulse wave forms were collected and images were analyzed using semiautomated edge tracking software (Artery Measurement System AMS) II version 1.141; Gothenburg, Sweden). Distensibility was calculated as the relative change in artery cross section (Eq. 7):

7 $$({\text{LD}}_{{{\text{max}}}} {-}{\text{LD}}_{{{\text{min}}}} )/({\text{LD}}_{{{\text{min}}}} \times {\text{PP}}))$$

using the maximum (LD_max_) and minimum (LD_min_) carotid artery lumen diameters in each heart cycle and the pulse pressure (PP) determined via pulse wave forms. Equation 8 was used to calculate compliance of the carotid artery:

8 $${\text{LD}}_{{{\text{max}}}} {-}{\text{ LD}}_{{{\text{min}}}} /{\text{PP}}$$

Equation (9) was used to calculate β-stiffness:

9 $${\text{ln}}\left( {{\text{SBP}}/{\text{DBP}}} \right)/\left[ {{\text{LD}}_{{{\text{max}} }} {-}{\text{ LD}}_{{{\text{min}}}} /{\text{LD}}_{{{\text{min}}}} } \right)$$

The same 10 heart cycles were used in the calculation for all three carotid stiffness outcomes.

### Statistical analysis

V̇O_2peak_ and Q̇_peak_ data were analyzed using a two-way mixed analysis of variance (ANOVA) with the between factor sex (2 levels; male vs. female) and within factor time (2 levels; baseline vs. 12 wk). A two-way mixed ANOVA (group × time) with 2 groups (male vs. female) and 3 time points (baseline vs. 4-wk follow-up vs. 12-wk follow-up) was performed to assess sex-based differences for changes in Hct, [Hb], FMD, and arterial stiffness (PWV, distensibility). In the presence of a significant group × time interaction, a Tukey’s multiple comparison’s post hoc test was used to compare changes in the outcome variables within each group (i.e., male and female). In the presence of a significant effect of time with no group × time interaction for variables measured at 3 time points, a Tukey’s multiple comparison’s post hoc test was used to compare changes in the outcome variables between the 3 time points combined across the groups (i.e., male and female not separated). An unpaired samples t-test was used to compare 1) V̇O_2peak_ relative to FFM, and 2) mean HR during SIT between males and females. Analyses were performed using GraphPad Prism 9 (GraphPad Software Inc, California, U.S.). Normality was tested using a Shapiro-Wilks test. Significance for all analyses was set to *p* ≤ 0.05. Results are presented as mean ± SD. Effect sizes were reported as η^2^_p_.

## Data Availability

Deidentified participant data that underlie the results presented in this article will be available to researchers upon review and approval of reasonable requests starting six months after manuscript publication. Proposals should be sent to the corresponding author.
